# Seamless Landsat-7 and Landsat-8 data composites covering all Amazonia

**DOI:** 10.1016/j.dib.2024.111034

**Published:** 2024-10-12

**Authors:** Rajit Gupta, Gabriela Zuquim, Hanna Tuomisto

**Affiliations:** a Department of Biology, University of Turku, 20014, Finland; b Department of Biology, Section for Ecoinformatics and Biodiversity, Aarhus University, Ny Munkegade 116, 8000 Aarhus C, Denmark

**Keywords:** Surface reflectance, Amazon forest, Google earth engine, BRDF correction, Pixel-based compositing

## Abstract

The use of satellite remote sensing has considerably improved scientific understanding of the heterogeneity of Amazonian rainforests. However, the persistent cloud cover and strong Bidirectional Reflectance Distribution Function (BRDF) effects make it difficult to produce up-to-date satellite image composites over the huge extent of Amazonia. Advanced pre-processing and pixel-based compositing over an extended time period are needed to fill the data gaps caused by clouds and to achieve consistency in pixel values across space. Recent studies have found that the multidimensional median, also known as medoid, algorithm is robust to outliers and noise, and thereby provides a useful approach for pixel-based compositing. Here we describe Landsat-7 and Landsat-8 composites covering all Amazonia that were produced using Landsat data from the years 2013–2021 and processed with Google Earth Engine (GEE). These products aggregate reflectance values over a relatively long time, and are, therefore, especially useful for identifying permanent characteristics of the landscape, such as vegetation heterogeneity that is driven by differences in geologically defined edaphic conditions. To make similar compositing possible over other areas and time periods (including shorter time periods for change detection), we make the workflow available in GEE. Visual inspection and comparison with other Landsat products confirmed that the pre-processing workflow was efficient and the composites are seamless and without data gaps, although some artifacts present in the source data remain. Basin-wide Landsat-7 and Landsat-8 composites are expected to facilitate both local and broad-scale ecological and biogeographical studies, species distribution modeling, and conservation planning in Amazonia.

Specifications Table

**Table:** 

Subject	Earth and Environmental Sciences
Specific subject area	Remote Sensing, Ecological and Biogeographical studies, Forestry
Type of data	Images: Satellite data imagery
Data collection	Landsat-7 Enhanced Thematic Mapper Plus (ETM+) surface reflectance (SR) image collection (LANDSAT/LE07/C01/T1_SR) and Landsat-8 Operational Land Imager (OLI) SR image collection (LANDSAT/LC08/C01/T1_SR) were processed in Google Earth Engine (GEE). The temporal period is the dry season of Amazonia (July–October) from 2013 to 2021 and applied a uniform cloud cover threshold of 60 %. Spatial resolution is 30 m. We utilized the three visible bands (Blue, Green, and Red), one near-infrared band (NIR), and two short-wave infrared bands (SWIR1 and SWIR2) from SR image collections.
Data source location	The dataset covers the spatial extent of Amazonia (Fig. 1) Spatial extent: Left −79.691W, Bottom: −20.494 S, Right: −43.398 W, Top: 9.589 N Coordinate Reference System (CRS): EPSG:4326 - WGS 84
Data accessibility	Dataset name: Amazonian Landsat 7 ETM+ and Landsat 8 OLI composites July to October 2013 to 2021 Repository name: Fairdata.fi Data identification number: https://doi.org/10.23729/e2eec23a-7549-49d0-9e95-765b79a8c14e Direct URL to data: https://etsin.fairdata.fi/dataset/703398d2-d03b-48b4-b35a-ac0634668028
Related research article	‘None’

## 1 Value of the Data

• Given the high incidence of clouds over Amazonia, it is difficult to produce clean Landsat composites that cover large areas of this rainforest biome. The Landsat 7 and Landsat 8 pixel-based seamless composites presented here have 30 m spatial resolution, and they are both gap-free and free of clouds and cloud shadows, as well as corrected for topographic and BRDF effects.
• The data are useful for a wide range of users in basic and applied sciences, including remote sensing experts, ecologists, conservatists and planners. The composites can be used as an input predictor layer in, for example, species distribution modeling, habitat mapping, the mapping of patterns in species composition or species richness, and machine and deep learning models for the prediction of biophysical and biogeographical variables.
• Many indices including Normalized Difference Vegetation Index (NDVI), Enhanced Vegetation Index (EVI) and Normalized Burn Ratio (NBR) can be easily derived by using bands from the Landsat composite data products. These indices are commonly used for the prediction of different response variables using modeling approaches.
• The production of properly pre-processed Landsat 7 and 8 image composites over all Amazonia requires considerable resources of memory and computing capacity. The datasets provide access to data where this work has already been done, and the accompanying GEE workflow makes it possible for users to create new composites using the resources provided by GEE.

## 2 Background

Amazonia is the largest tropical forest area in the world. However, Amazonia is poorly known and difficult to access, which is why remote sensing plays a crucial role to understand its ecology and heterogeneity [1]. Landsat data can facilitate the mapping of Amazonian rainforest characteristics and thereby generate products with significant implications for scientific community and policymakers [2]. The wet rainforest climate causes many images over Amazonia to be cloudy, such that producing a cloud-free composite necessitates compiling imagery over a relatively long time period [3]. A second challenge is the bi-directional reflectance distribution function (BRDF) effect, which refers to different scattering of light in different parts of the same Landsat scene due to varying relative positions of sun, object and sensor [4,5]. Moreover, the sheer size of Amazonia means that high processing resources are required to cover the Amazon basin or even a substantial part of it. The existing basin-wide Landsat composites provide snapshots in time, but many practical applications would benefit from having access to more recent composites. Additionally, the existing composites may not have the desired quality over the area of interest, and a longer compositing period might be needed to reduce data gaps or atmospheric effects.

## 3 Data Description

**Fig. 1:**
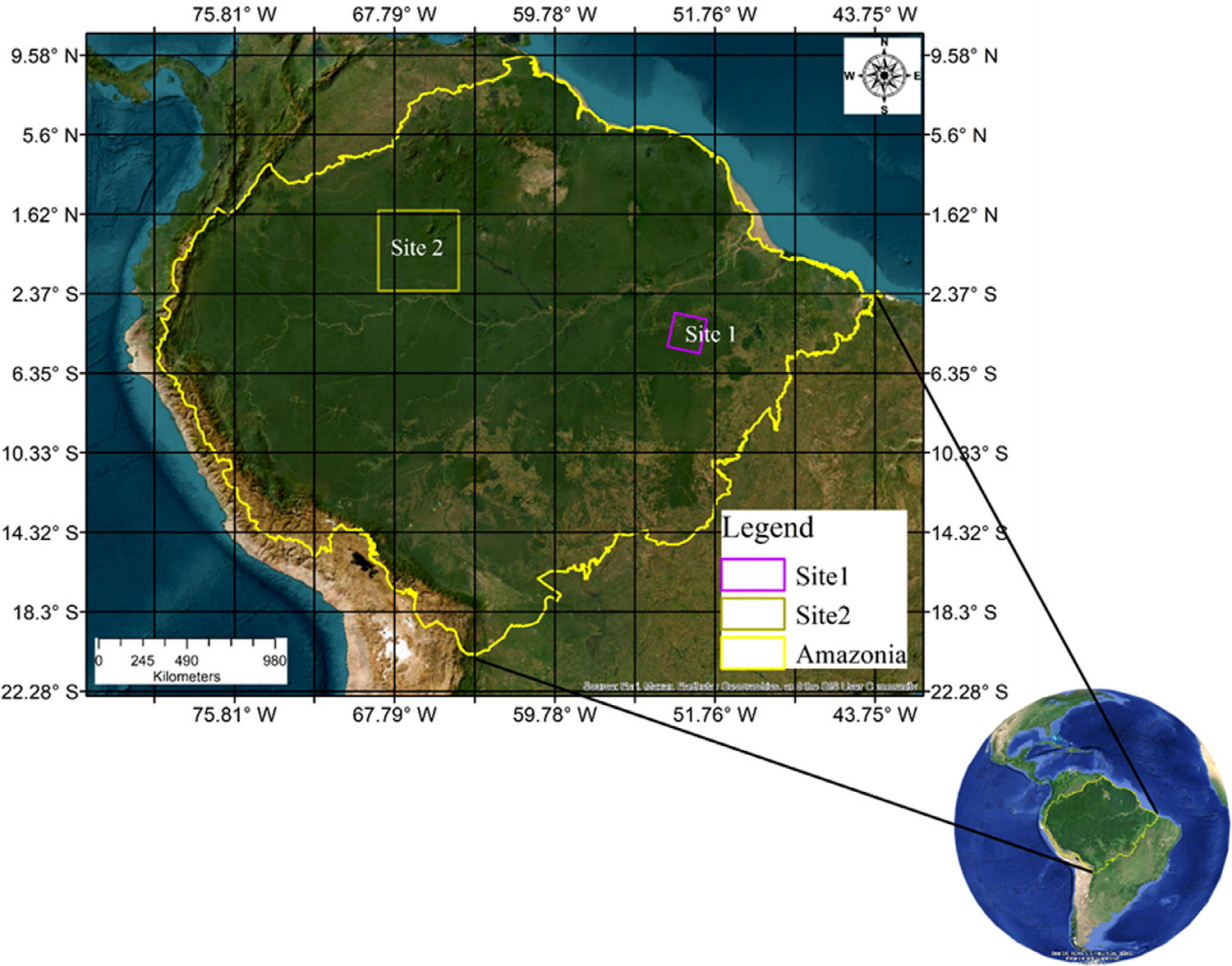
Spatial extent of Amazonia as used in this study. The Landsat 7 and Landsat 8 composites cover the entire area within the yellow line and are distributed in tiles as indicated by the grid. Numbered squares indicate focal areas that were used for visual inspection of Landsat composite quality. (For interpretation of the references to color in this figure legend, the reader is referred to the web version of this article.)

Each six-band Amazon-wide composite is distributed in about 4° by 4° tiles at 30-m spatial resolution (Fig. 1). The data repository has a main folder "Landsat 7 and 8 composite" that contains a separate subfolder for each composite, named “Landsat_7_30m_tiles” and “Landsat_8_30m_tiles”. Both folders are 261 GB in size and contain 56 tiles. Filenames contain either L7 (Landsat 7) or L8 (Landsat 8) in addition to coordinates representing a bounding box (X-min, Y-min, X-max, Y-max) of the tile. Each tile is up to 4.89 GB in size, with areas outside the limiting boundary of Amazonia clipped. Tiles are in tiff format, and after downloading the data can be opened using software like QGIS, ArcMap, RStudio, Jupyter notebook etc. Each tile contains six bands: Band 1, Band 2, Band 3, Band 4, Band 5 and Band 6, corresponding to Blue, Green, Red, NIR, SWIR 1 and SWIR2, respectively. The Coordinate Reference System (CRS) is EPSG:4326 - WGS 84.

## 4 Experimental Design, Materials and Methods

### 4.1 Study area

Our main interest is in the Amazonian lowland rainforest biome, but for the purposes of this study, we used a broader area delimitation that includes all Andean areas that drain into the Amazon river, as well as the Guianas and the forested parts of the Orinoco river drainage. The boundary limit was combined from available source polygons [[6], [7], [8]] and it covers a total surface area of approximately 6.7 million square kilometers (Fig. 1).

### 4.2 Data collection

We used Landsat-7 Enhanced Thematic Mapper Plus (ETM+) surface reflectance (SR) image collection (LANDSAT/LE07/C01/T1_SR) and Landsat-8 Operational Land Imager (OLI) SR image collection (LANDSAT/LC08/C01/T1_SR). Products from these two sensors differ in both spectral wavelength and radiometric resolution. The Landsat-7 ETM+ has 8 bits while Landsat 8 OLI has 12 bits of radiometric resolution. We utilized the three visible bands (Blue, Green, and Red), one near-infrared band (NIR), and two short-wave infrared bands (SWIR1 and SWIR2) to produce six-band Landsat-7 and Landsat-8 composites over Amazonia. In Landsat 7, these correspond to bands 1–6 and 7, but in Landsat 8 they correspond to bands 2–7. We used SR images from GEE acquired between September 2013, and October 2021 [9]. To avoid excessive processing of cloudy images and noise contamination, only data from the four driest months were used (July to October).

### 4.3 Landsat data pre-processing

**Fig. 2:**
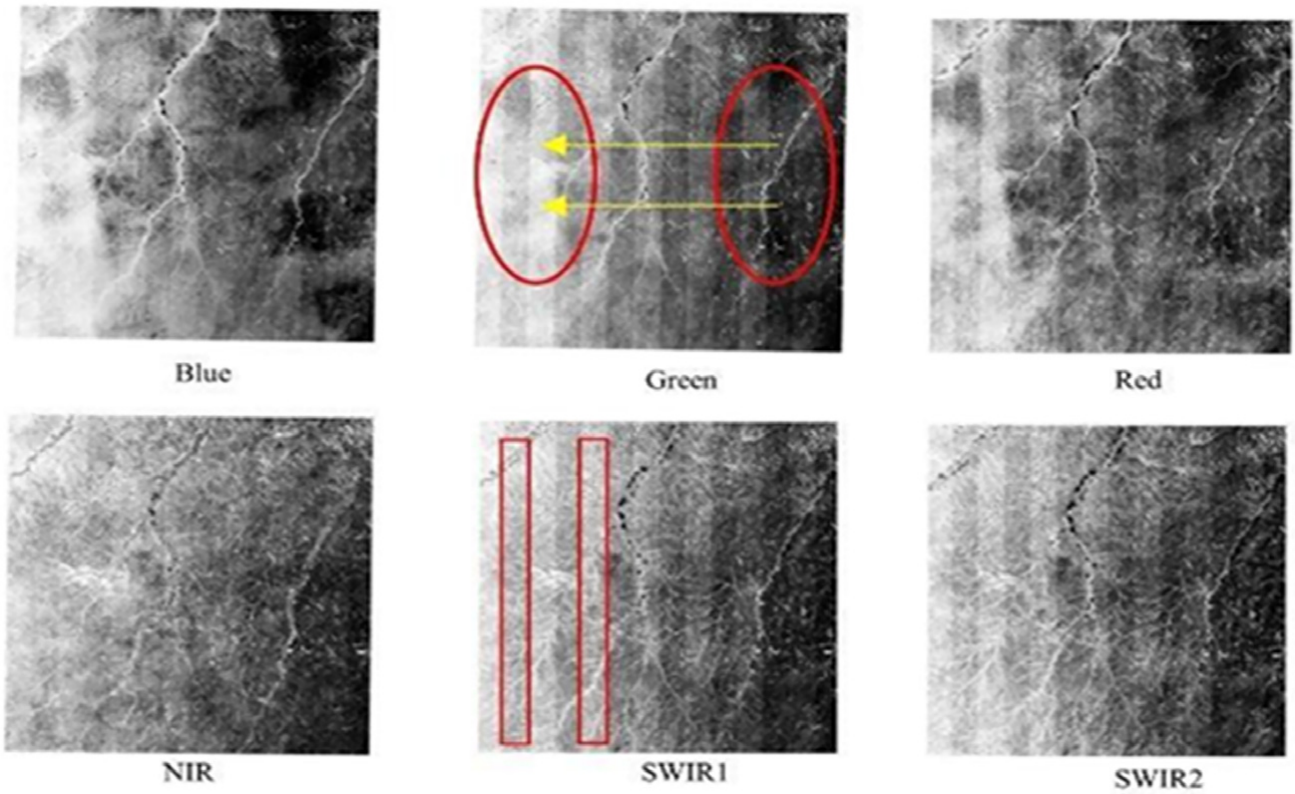
Example of raw Landsat-8 data SR image with each panel corresponding to a different wavelength band. Uncorrected BRDF effects cause a black in the east to white in the west gradient (highlighted on the panel corresponding to the Green band). Landsat-8 data also suffer from even/odd artifactual striping due to sensor miscalibration (highlighted on the SWIR1 band). (For interpretation of the references to color in this figure legend, the reader is referred to the web version of this article.)

**Fig. 3:**
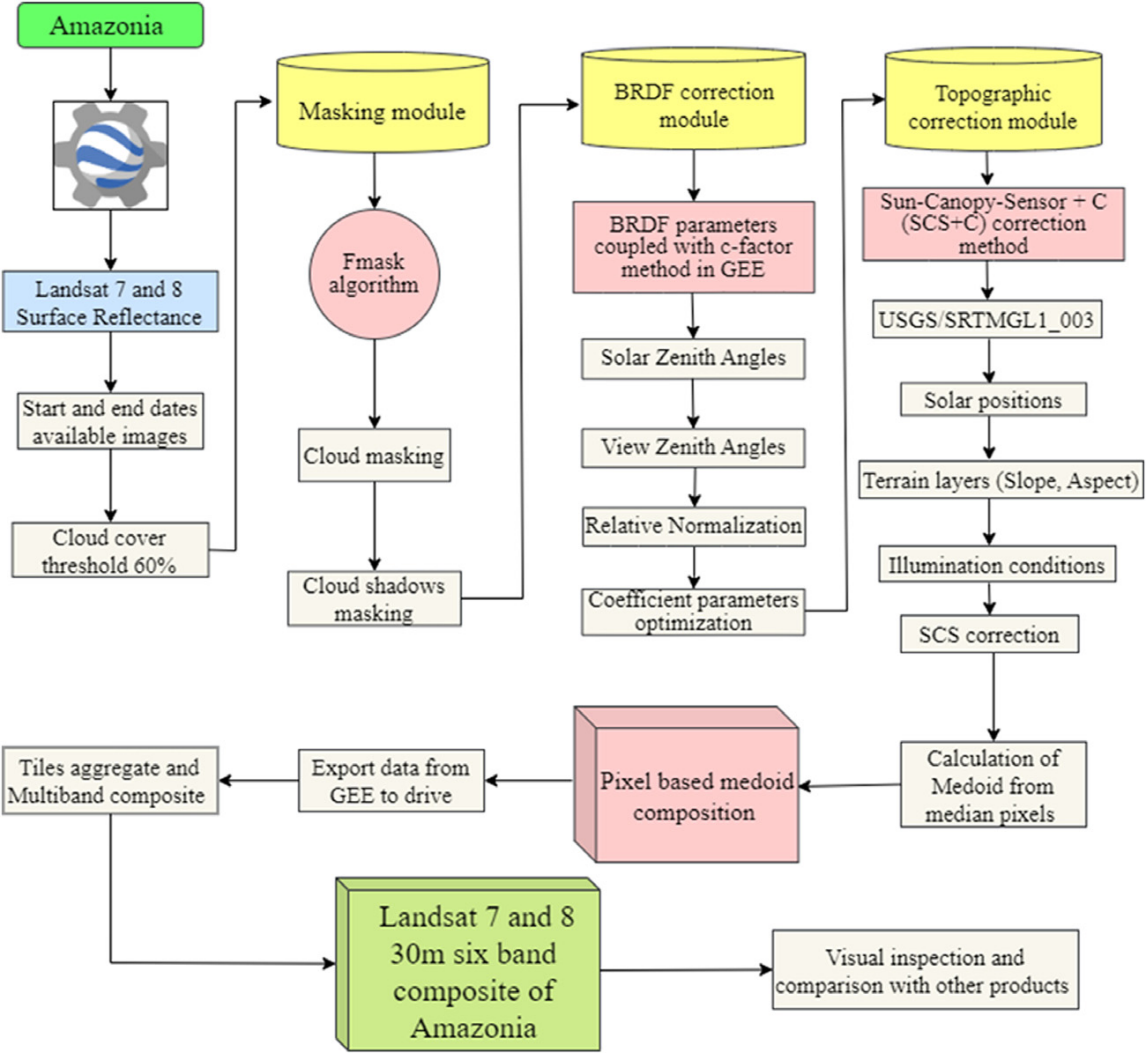
The methodological framework used in this study.

The pre-processing setup consisted of three modules for the atmospherically corrected Landsat-7 and Landsat-8 data in GEE. The first one masked clouds and cloud shadows, the second one corrected for brightness differences caused by shadows of hills, and the third one removed BRDF effects. In addition, each sensor has specific problems that could not be remedied. Landsat 7 has a scan line error, which leads to missing data in a subset of the lines, and Landsat 8 has even-odd calibration problems that result in alternate higher and lower reflectance bands (Fig. 2) [10,11]. Our detailed methodological framework is shown in Fig. 3.

### 4.4 Pixel-based medoid compositing

Various techniques have been developed for the compositing of multi spatio-temporal images. We used the medoid compositing method, which has been found to perform well in earlier studies [3]. This algorithm takes all valid and unmasked observations for each pixel, calculates their median value for each wavelength band, and chooses the observation that is closest to this medoid in n-dimensional spectral space [12]. The algorithm produces composites that are robust to outliers, have reduced noise and are radiometrically consistent [3]. Our entire workflow can be reproduced both for Landsat-7 data (https://code.earthengine.google.com/4d006465df9447c7be426b87b612ab96) and for Landsat-8 data (https://code.earthengine.google.com/a475248819b9392d95fa4217dac7296b).

### 4.5 Visual inspection

**Fig. 4:**
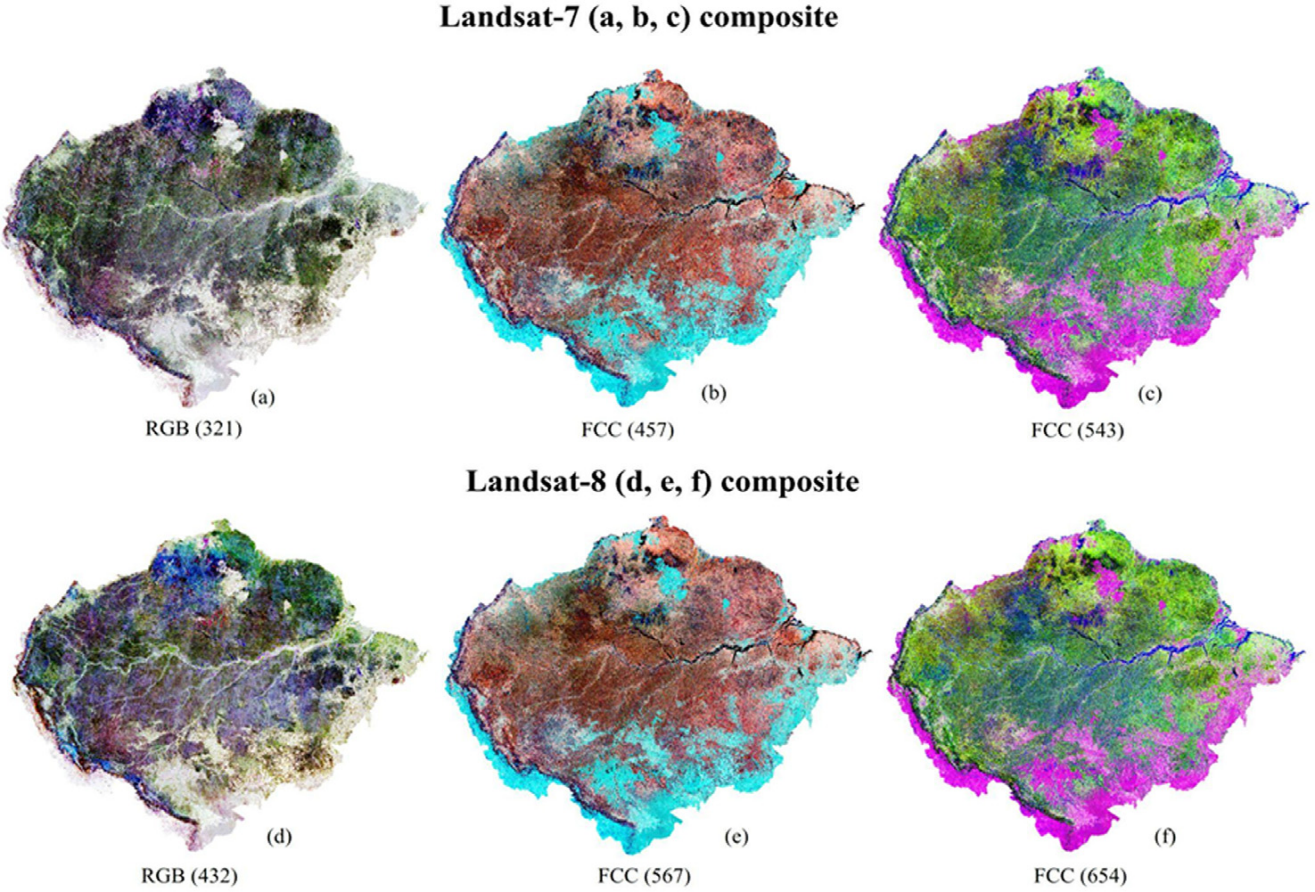
Amazon-wide composites of Landsat-7 data (top row) and Landsat-8 data (bottom row). True color composites with the Landsat red, green and blue bands (numbered 3, 2 and 1 in Landsat-7 and 4, 3 and 2 in Landsat-8) assigned to red, green and blue, respectively (a, b). False color composites with the Landsat NIR, SWIR1 and SWIR2 bands (numbered 4, 5 and 7 in Landsat-7 and 5, 6 and 7 in Landsat-8) assigned to red, green and blue, respectively (b, e). False color composites with the Landsat SWIR1, NIR and red bands (numbered 5, 4 and 3 in Landsat-7 and 6, 5 and 4 in Landsat-8) assigned to red, green and blue, respectively (c, f). (For interpretation of the references to color in this figure legend, the reader is referred to the web version of this article.)

**Fig. 5:**
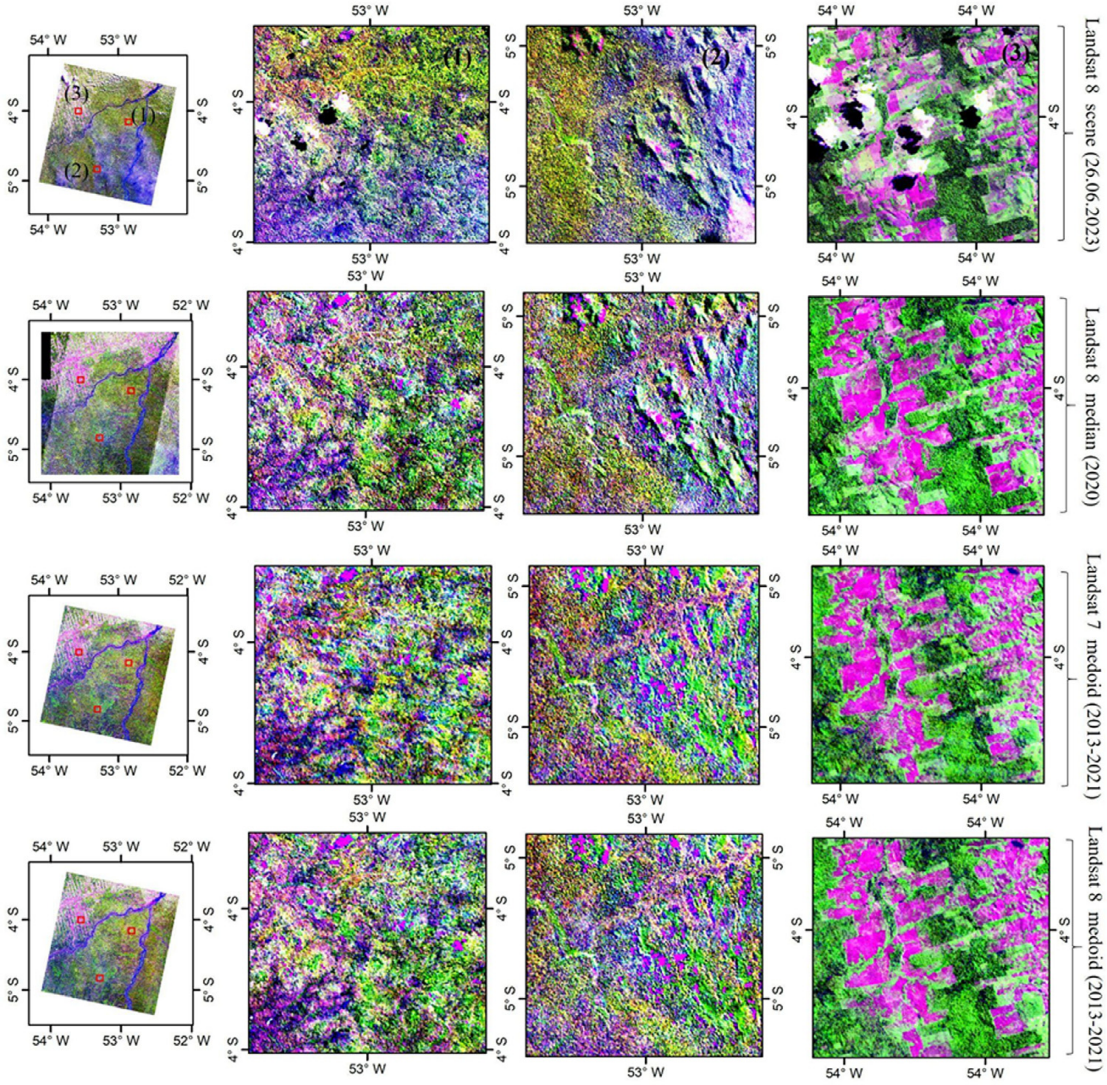
Visual comparison of details in different Landsat products at a local scale (site 1) (see Figure 1 for the location within Amazonia). Top row from a single Landsat-8 scene; second row a one-year composite from MapBiomas, bottom two rows the new 9-year Landsat composites from the present study. All windows show false color composites with SWIR1, NIR and red bands (numbered 5, 4 and 3 in Landsat-7 and 6, 5 and 4 in Landsat-8) assigned to red, green and blue, respectively. (For interpretation of the references to color in this figure legend, the reader is referred to the web version of this article.)

**Fig. 6:**
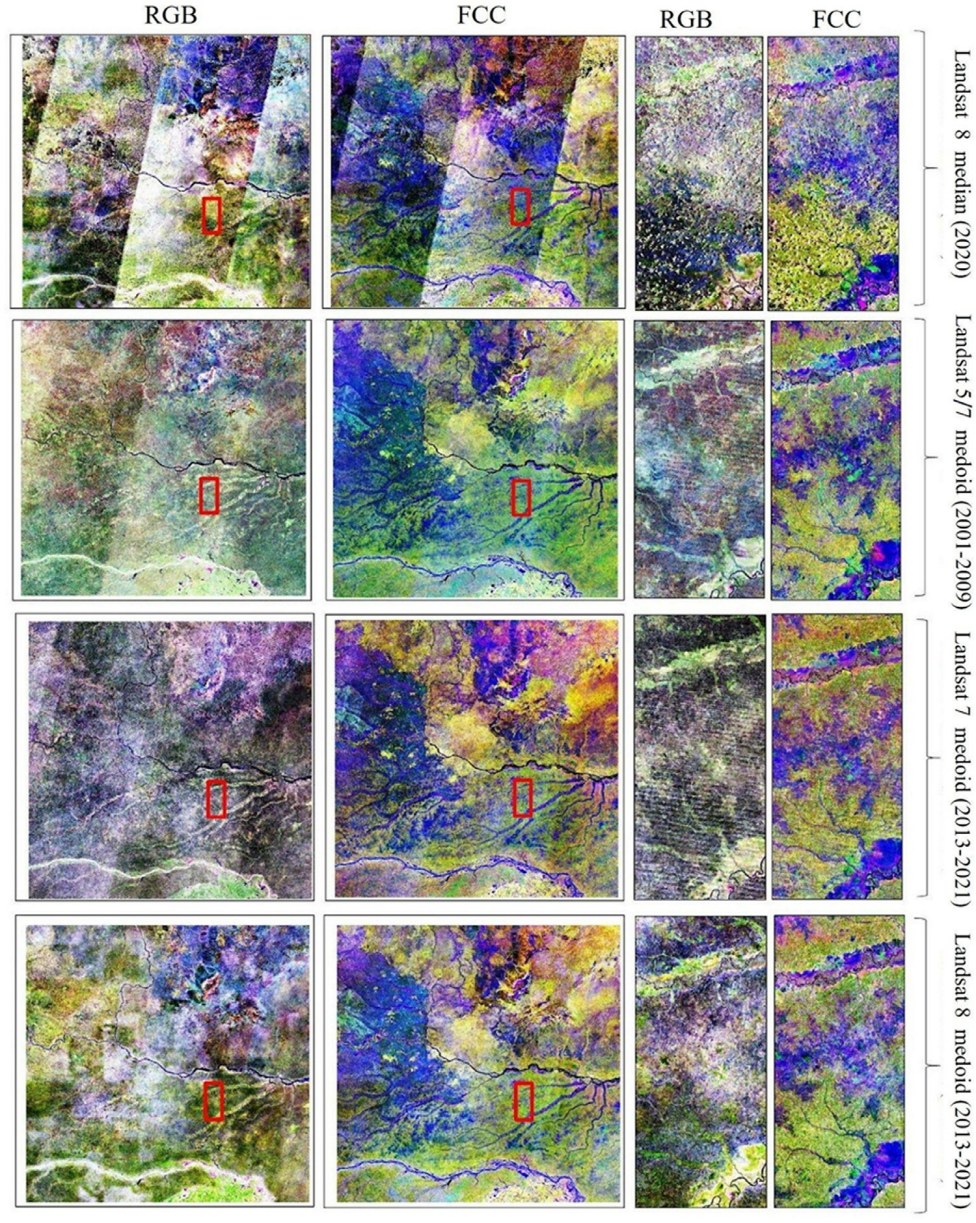
Visual comparison of earlier Landsat composites (top row from MapBiomas; second row from Van doninck and Tuomisto, 2018) with those produced in the present paper (bottom two rows) at a regional scale (site 2) (see Fig. 1 for the location within Amazonia). Panels with true color composites (RGB) (numbered 3, 2 and 1 in Landsat-7 and 4, 3 and 2 in Landsat-8) assigned to red, green and blue, respectively. Panels with false color composites (FCC) (numbered 5, 4 and 3 in Landsat-7 and 6, 5 and 4 in Landsat-8) assigned to SWIR1, NIR, and red, respectively. (For interpretation of the references to color in this figure legend, the reader is referred to the web version of this article.)

The Amazonian Landsat-7 and Landsat-8 composites with different band combinations are shown in Fig. 4 (one true color and two false color composites). To visually assess the quality of our Landsat image composites, we compared them with other Landsat products. First, we compared them with the already existing basin-wide Landsat composite produced by using 16,000 Landsat-5 and Landsat-7 images of the years 2000–2009 [5]. In addition, we focused on two smaller areas in different parts of the Amazon basin (squares in Fig. 1). For site 1, we downloaded a single Landsat-8 data scene with minimum cloud cover (<10 %; 26 June 2023) from the United States Geological Survey (USGS) EarthExplorer [13]. For site 2, we produced annual median composites for the year 2020 using the toolkit provided by MapBiomas in GEE [8]. Visual evaluation of the composites and comparison with other products confirmed that the corrections worked well, although both Landsat-7 and Landsat-8 data have technical issues that need to be taken into account when using them for specific applications (Fig. 5, Fig. 6).

## Limitations

Creating pixel-based medoid composites for large areas like the Amazon with proper corrections requires significant computation and memory resources. GEE has limited capability and memory, so Amazonia was divided into small tiles for composite creation, which were then aggregated into a full Amazonian composite. Landsat-7 has scan line errors and reflectance differences between adjacent flight paths, while Landsat-8 has even/odd striping caused by sensor miscalibration. Comparing Landsat-7 and Landsat-8 medoid composites shows no clear winner for practical applications, as both have their issues and advantages, varying by region and study resolution.

## Ethics Statement

The authors have read and follow the ethical requirements for publication in Data in Brief and confirming that the current work does not involve human subjects, animal experiments, or any data collected from social media platforms.

## CRediT Author Statement

**Rajit Gupta:** Conceptualization, Software, Methodology, Data curation, Visualization, Formal analysis, Writing – original draft. **Gabriela Zuquim:** Software, Writing - review and editing. **Hanna Tuomisto:** Conceptualization, Funding acquisition, Investigation, Supervision, Writing - review and editing.

## Data Availability

Fairdata.fiAmazonian Landsat 7 ETM+ and Landsat 8 OLI composites July to October 2013 to 2021 (Original data)
